# Inhibition of Predator Attraction to Kairomones by Non-Host Plant Volatiles for Herbivores: A Bypass-Trophic Signal

**DOI:** 10.1371/journal.pone.0011063

**Published:** 2010-06-10

**Authors:** Qing-He Zhang, Fredrik Schlyter

**Affiliations:** Chemical Ecology, Department of Plant Protection Biology, Swedish University of Agricultural Sciences, Alnarp, Sweden; INRA - Paris 6 - AgroParisTech, France

## Abstract

**Background:**

Insect predators and parasitoids exploit attractive chemical signals from lower trophic levels as kairomones to locate their herbivore prey and hosts. We hypothesized that specific chemical cues from prey non-hosts and non-habitats, which are not part of the trophic chain, are also recognized by predators and would inhibit attraction to the host/prey kairomone signals. To test our hypothesis, we studied the olfactory physiology and behavior of a predaceous beetle, *Thanasimus formicarius* (L.) (Coleoptera: Cleridae), in relation to specific angiosperm plant volatiles, which are non-host volatiles (NHV) for its conifer-feeding bark beetle prey.

**Methodology/Principal Findings:**

Olfactory detection in the clerid was confirmed by gas chromatography coupled to electroantennographic detection (GC-EAD) for a subset of NHV components. Among NHV, we identified two strongly antennally active molecules, 3-octanol and 1-octen-3-ol. We tested the potential inhibition of the combination of these two NHV on the walking and flight responses of the clerid to known kairomonal attractants such as synthetic mixtures of bark beetle (*Ips* spp.) aggregation pheromone components (*cis*-verbenol, ipsdienol, and *E*-myrcenol) combined with conifer (*Picea* and *Pinus* spp.) monoterpenes (α-pinene, terpinolene, and Δ^3^-carene). There was a strong inhibitory effect, both in the laboratory (effect size *d* = −3.2, walking bioassay) and in the field (*d* = −1.0, flight trapping). This is the first report of combining antennal detection (GC-EAD) and behavioral responses to identify semiochemical molecules that bypass the trophic system, signaling habitat information rather than food related information.

**Conclusions/Significance:**

Our results, along with recent reports on hymenopteran parasitoids and coleopteran predators, suggest that some NHV chemicals for herbivores are part of specific behavioral signals for the higher trophic level and not part of a background noise. Such bypass-trophic signals could be of general importance for third trophic level players in avoiding unsuitable habitats with non-host plants of their prey.

## Introduction

Insect predators and parasitoids exploit a variety of chemical signals from different trophic levels as kairomones and synomones to locate their herbivorous prey and hosts in tri-trophic systems [1]–[4]. These attractive chemical signals (behavioral chemicals = semiochemicals) may include pheromones of herbivores (second trophic level), host plant kairomones of herbivores (first trophic level), and herbivore-induced plant odor synomones (combination of first and second trophic levels). Behavioral responses to kairomones (positive signals) lead natural enemies to suitable breeding sites and habitats, as well as ensuring encounter with mates and availability of prey and/or hosts. The importance of these positive signals has been widely documented and accepted [2], but the potential role of negative signals (behavioral inhibitors, interruptants, or repellents) from non-prey and non-host habitats has rarely been studied [1], [4], [5].

Conifer bark beetles not only detect and orient to their aggregation pheromone and host volatiles, but also are able to perceive and respond behaviorally to volatiles from non-host angiosperm trees [6], [7]. For instance, the Eurasian spruce engraver, *Ips typographus* (L.) (Coleoptera: Scolytidae), can recognize and avoid three specific alcohols from green leaves (1-hexanol, (*Z*)-3-hexen-1-ol, and (*E*)-2-hexen-1-ol = GLV); two C_8_-alcohols (3-octanol and 1-octen-3-ol); and a spiroacetal *trans*-conophthorin from angiosperm bark [8]–[10]. Such specific olfactory recognition and inhibitory behavioral effects on attraction of angiosperm non-host volatiles (NHV) have been reported for several other conifer bark beetle genera in both Eurasia and in North America [7], [11]. In several insects orders, the inhibitory effects of NHV at second trophic level are reported: Coleoptera [7], [12], Diptera [13], Homoptera [14], and Lepidoptera [15].

The checkered beetle, *Thanasimus formicarius* (L.) (Coleoptera: Cleridae), is a common predator of European conifer bark beetles, such as the pine shoot beetle, *Tomicus piniperda* (L.), and *I. typographus* [16], [17]. Its prey range is mostly restricted to conifer bark beetles [18], and there are reports of a reduced prevalence of the clerid in broad-leaf or mixed forest compared to more pure spruce or pine stands [19]–[21]. The checkered beetle is attracted to suitable trees by the same volatiles that bark beetles use for locating host trees and their mates [22]. The volatiles are conifer host monoterpenes for *To. piniperda* [23], and aggregation pheromone components in *I. typographus* and the striped ambrosia beetle, *Trypodendron lineatum* (Olivier) [24], [25]. Physiological evidence from electroantennography (EAG) and from single cell recordings (SCR) show that *T. formicarius* has olfactory receptor cells specialized to bark beetle pheromone components and to prey host plant volatiles, with sensitivity and specificity similar to that of its prey [26], [27].

**Figure 1:**
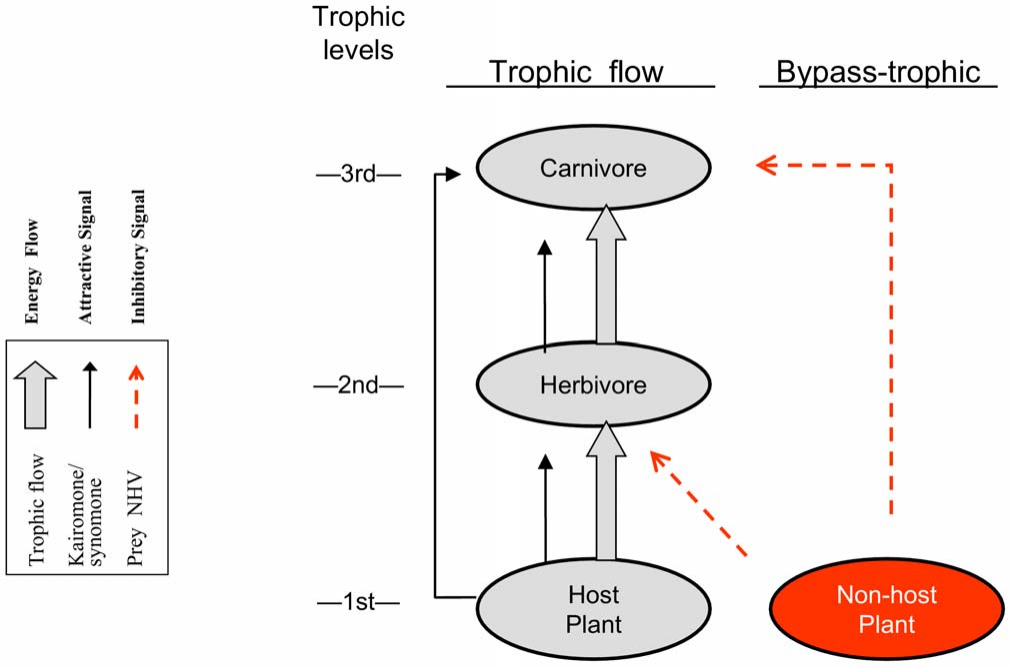
Semiochemical signals involved at various trophic and/or bypass-trophic levels. Trophic levels and flows are coded by gray fill and solid arrows give the trophic semiochemical signal flow (kairomone from plants at 1^st^ trophic level and from herbivores at 2^nd^ level). The corresponding signals that bypass the trophic flow are dashed. White text, red fill: The trophic level besides the flow of energy and matter, the non-host plant. The 3^rd^ carnivore level corresponds here to the clerid beetle, *Thanasimus*.

However, based on optimal foraging theory [28] and the pervasiveness of NHV in conifer bark beetle systems [7], we propose the following hypothesis: *insect predators (and parasitoids) are able to recognize their prey's non-host plants and habitats by using specific semiochemicals, e.g., NHV components* (Figure 1). Such cues would not be multi-trophic [4] or trophic, as there is no direct trophic relation between a predator and the non-host of its prey. Instead, the proposed semiochemical signals would bypass the trophic or food chains. In a mixed habitat, with few host plants available, a kairomone signal from the herbivore could be masked by the non-host volatiles, which may represent a form of prey escape.

The close association and similarity in olfactory perception between the conifer bark beetles and their checkered beetle predators allow us to use *T. formicarius* as a model insect to test our hypothesis. As a starting point, we investigated antennal activity of the predator to compounds ecologically relevant to either the prey or the predator, followed by behavioral tests of those antennally active in the predator. We predict that in the predator there is specific olfactory recognition and inhibitory behavioral effects on attraction to kairomone of some semiochemical molecules from the non-host plants of its prey.

## Results

### Antennal responses

**Figure 2:**
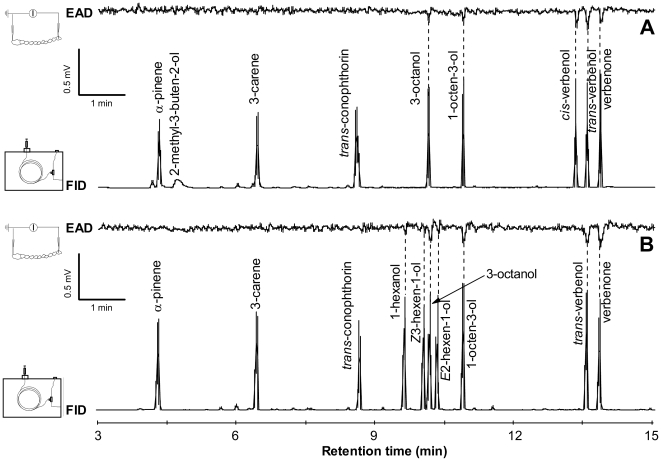
GC-EAD responses of predator antennae to synthetic kairomone blends reveal strong activity of C_8_ alcohols. Both blends contain conifer tree volatiles (α-pinene and Δ^3^-carene), general bark beetle pheromone components (*trans*-verbenol and verbenone) and some volatiles from trees not exploited by the prey of the clerids (NHV for the prey) from angiosperm bark (C_8_-alcohols and *trans*-conophthorin). For each compound, ca. 100 ng was injected. Thus after splitting (1∶1), ca. 50 ng of each compound passed over each *Thanasimus formicarius* antennal preparation. Vertical dashed lines connect peaks from flame ionization detection (FID) with repeatable peaks from electrographic antennal detection (EAD). A) The base blend plus 2-methyl-3-buten-2-ol and *cis*-verbenol, main components of the *Ips typographus* pheromone; B) The base blend plus the GLV (1-hexanol, (*Z*)-3-hexen-1-ol, and (*E*)-2-hexen-1-ol), which are active in behavioral inhibition of the prey, *I. typographus*.

The antennae of *T. formicarius* gave consistent and strong responses, not only to the common bark beetle pheromone components, *cis*-verbenol, *trans*-verbenol, and verbenone (Figure 2A), but also to two volatiles from trees not exploited by the prey of the clerids (i.e. NHV for the prey). The two C_8_-alcohols, 3-octanol and 1-octen-3-ol (Figure 2), elicited responses in five of five preparations for each sex in the GC-EAD analysis of our two similar synthetic mixtures. Weaker but repeatable responses were also detected to the three GLV in ca. 50% of EAD recordings (Figure 2B). However, at the doses (ca. 50 ng) tested, no responses were recorded to some of the compounds that are highly relevant to herbivores, such as 2-methyl-3-buten-2-ol (a pheromone component of *I. typographus*), and the two conifer monoterpenes, α-pinene and Δ^3^-carene (kairomone components for *To. piniperda*). Surprisingly, one of the most antennally- and behaviorally-active NHV for conifer bark beetles, *trans*-conophthorin, elicited no antennal depolarization in the clerids at the dose tested (Figure 2). There were no differences in the frequency of repeatable EAD responses or in signal amplitude between the sexes.

### Walking bioassay

**Figure 3:**
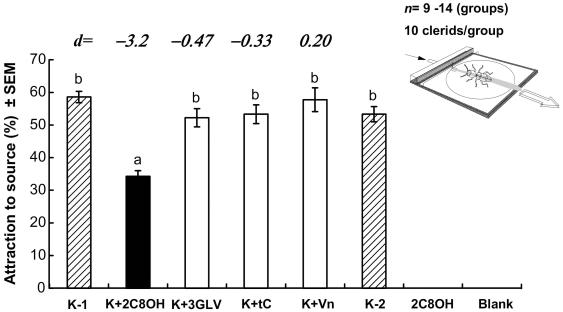
Responses of the walking predator in an olfactometer show inhibition of attraction by C_8_ alcohols. Mean responses (±SEM) of *Thanasimus formicarius* to various semiochemical treatments in a laboratory open-arena walking assay. Treatments included the attractant kairomone (K-1 as 1^st^ test of positive control before testing on any inhibitor candidates; K-2 as 2^nd^ test of the same positive control after inhibitor-related treatments) alone and in combination with potential behavioral inhibitors (Table 2). Abbreviated treatments are: **K**) Kairomone blend [MB/cV/Id/aP, see Table 2]; **2C8OH**) blend 1∶1 of two bark C_8_-alcohols (3-octanol+1-octen-3-ol); **3GLV**) 1-hexanol, (*Z*)-3-hexen-1-ol and (*E*)-2-hexen-1-ol; and **Vn**) verbenone. Bars with the same letter are not significantly different (*P*>0.05) by ANOVA, followed by REGW-Q test. The blank control and the 2C8OH treatment (with zero responses) were not included in the ANOVA and the range tests to achieve homogeneity of variances. There were *n* = 14 runs of 10 *T. formicarius* beetles for Kairomone blend and 2C8OH; *n* = 9 runs for all other stimuli. Numbers in italicized font above bars are standardized effect sizes [29], as bias corrected Hedges' *d* [75], [76], see Statistics. Negative values show a reduction of attraction response. For clarity, the clerid beetle is drawn at ×15 larger scale than arena (insert).

Approximately 60% of *T. formicarius* adults were attracted in the walking bioassay to the first kairomone source presented (Figure 3). When a blend of the two C_8_-alcohols was added to the kairomone source, the attractive response was significantly reduced to 34% (ANOVA, F_5,58_ = 16.75; *P*<0.05; Figure 3). The effect size for the combination of the two C_8_-alcohols (*d* = −3.2) was quite large. Conventionally, effect sizes of 0.5 are regarded as ‘medium’ and ≥0.8 as ‘strong’ [29]. Addition of the GLV mixture, *trans*-conophthorin, or verbenone did not significantly affect the attraction and the effect sizes of these NHV for the prey were medium to small (*d* from −0.47 to 0.20). A second test of the kairomone alone showed no decline in response during testing. Neither the blank control nor the blend of the two C_8_-alcohols attracted any clerids (Figure 3).

### Field trapping

In experiment 1 (May 7 to 11, 2000) the addition of the two C_8_-alcohols to the kairomone attractant significantly reduced the catches of *T. formicarius* by more than 36% (Table 1).

**Table 1:** Catches of *Thanasimus* predator and *Ips* prey in the multiple funnel/barrier trap groups baited with either kairomone alone or kairomone plus different NHV components for the prey, Småland, Sweden.

Experiment	Treatment†	*Thanasimus formicarius*	*Ips typographus*
		(Mean±SE)	(Mean±SE)
**Experiment 1 (** ***n*** ** = 6)**			
	Kairomone	11.5±1.5^a^	133±19.3^a^
	Kairomone +C_8_-alcohols	7.33±1.73^b^	93.0±10.6^b^
Effect size*, ***d***		***−0.97***	***−0.96***
**Experiment 2 (** ***n*** ** = 7)**			
	Kairomone	31.1±9.57^a^	276±94.7^a^
	Kairomone +3GLV+tC+Vn	30.0±8.94^a^	147±76.5^b^
Effect size, *d*		***−0.05***	***−0.49***

†)**Kairomone** composed of the pheromone components of *Ips typographus* and *I. duplicatus* plus the host attractants of *Tomicus piniperda*, three of the major bark beetle prey species in Scandinavia. In all 2+2+3 = 7 kairomone components, for details of compounds and dispensers, see Table 2, part 2. **NHV components for the prey (C8, 3GLV, tC, Vn)**; for abbreviations and details of the compounds and their dispensers, see Table 2, part 2.

a)Values with the same letter in each column and experiment are not significantly different within the experiment (*P*>0.05) by paired *t*-test. *n* = trap pair rotations.

*)Standardized effect size, as bias corrected Hedges' *d* [76]. Negative values show a reduction of attraction response measured as the trap catch. Conventionally, effect sizes of 0.5 are regarded as ‘medium’ and ≥0.8 as ‘strong’ [29].

Catches of *I. typographus* were similarly lower to the treatment with the C_8_-alcohols. The effect sizes were not only relatively large for the combination of the two C_8_-alcohols, they were also quite similar for both predator and prey responses (*d* for both≈−1.0, Table 1). Correspondingly, the ratios of predator to prey in both treatments were almost the same (1∶11 −12). No differences in trap catch of *T. formicarius* were found in experiment 2 (May 12 to June 26, 2000) when the blend of GLV, *trans*-conophthorin, and verbenone (compounds with weak EAD-activity in the laboratory), was added to the kairomone source. In contrast, catches of *I. typographus* were reduced by nearly 50% (*d* = −0.49) relative to those in the kairomone-baited positive control trap (Table 1). Thus, one effect of the treatment was to double the predator/prey ratio. In both experiments, we trapped another clerid species, *T. femoralis* (Zett.), in low numbers. The response pattern was similar to that of *T. formicarius* [24], [30], but the numbers caught did not permit for any further analyses. The two other bark beetles whose attractants we used in the kairomone blend, *I. duplicatus* and *Tomicus* spp, were not caught as trapping was done outside their distribution area or flight periods, respectively.

## Discussion

Our electrophysiological and behavioral data show that the checkered beetle can detect not only olfactory signals directly from the trophic chain, e.g., kairomonal compounds such as prey pheromone components or host plant volatiles of its prey, but also specific volatiles from trees not exploited by the prey of the clerids (Figure 1). This clerid predator of conifer bark beetles thus responds to a subset of the angiosperm semiochemicals, recognized also by their herbivore prey (NHV for the prey).

Therefore, the signal recognized by the predator is not strictly related to the food chain, but it originates from the first trophic level (plants not fed upon by the herbivore) and bypasses the 2^nd^ trophic level to be effectively used by the 3^rd^ level (Figure 1). We designate such an anti-attractant signal used by the predator a “bypass-trophic signal” since it passes to the side the trophic flow of material and energy; benefiting the receiver but having no positive or negative effects on the emitter (Figure 1). Earlier proposed terms for multi-trophic relations [31], like ‘synomone’, require a benefit to both receiver and sender; the sender (a plant) is here not directly involved with the receiver, and thus does not benefit from the response to it by the carnivore. Although, the terms like ‘synomone’ [31] or ‘enemy avoidance kairomone’ [32] do include signals that oppose attraction, they are not applicable here, as they focus only on the selective values for emitter and receiver that are linked directly in a bi- or tri-trophic chain.

However, alternative interpretations are possible: our collection methods may have biased the preferences displayed in the laboratory bioassay and the total number of active compounds is probably higher than the two we have identified.

That we collected predators hunting on conifer wood log piles may have introduced a bias in the walking bioassay towards a preference of these collected insects for conifer odors. Such a bias could be due to learning (association with prey capture) or sub-sampling effects (from a conifer only habitat). The subsequent field test, however, relied on effects on the attraction of insects from a freely dispersing natural population from a forest landscape with both conifer and mixed forests [33].

Volatiles in the forest habitat are many and compounds other than the two found by us may well be detectable by the predator. More or less ecologically relevant volatiles could act as components of ‘noise’ and further modify predator behavior towards attractive signals. Little is known regarding the mechanisms of a 3^rd^ level player in olfactory biology [27], [34], but there are limitations on the number of molecules detectable by insects. While insects have a highly sensitive and specific olfactory sense, their sense is constrained by the range of compounds specifically detected, probably due to their small body size. Insect long-range responses to plant odors are not based on “generalist” neurons responding to many similar molecules of “general plant compounds”, but by highly specific and sensitive responses from sensory cells to single key compounds, from host or non-host plants or other sources [27], [35]–[38]. Habitat odors are typically present in quite low amounts [39]. Bark beetles or their predators need to have specific olfactory receptor neurons on their antennae for these natural volatile chemicals normally present in low amounts, to be able to detect, recognize, and respond to them behaviorally.

The response pattern and sensitivity to these semiochemicals exhibited by *T. formicarius* are specific and clearly different from those shown by a major prey species, *I. typographus*. For instance, the non-host plant volatiles that were most active in reducing attraction in the checkered beetle, the two C_8_-alcohols (3-octanol and 1-octen-3-ol), were among the NHV showing weak antennal activity in *I. typographus*. Conversely, the GLV (C_6_-alcohols) from non-host leaves and partly from bark, which are strongly active in many conifer bark beetles [7], showed repeatable but weak antennal responses and were not active in the field for the predator at the release rates tested. *trans*-Conophthorin is the most active NHV for several *Ips* bark beetles in the *T. formicarius* prey range [7], [40], but showed no signs of activity in this clerid at the release rate tested. However, the lack of physiological detection and behavioral response to *trans*-conophthorin has also been reported for several conifer bark beetle species, including some *T. formicarius* prey species like the pine shoot beetles *To. piniperda* and *To. mino*r [41]. The most active individual NHV components and blends vary also for different scolytid species, but the reason for the variation is far from understood [7], [11].

In addition to their occurrence in angiosperm trees [8], [42], the two C_8_-alcohols have been reported from a wide range of natural sources. These alcohols are found in volatiles emitted by fungi [43]–[45], cucujid grain beetles [46], mammals [47], fruit [48], beans [49], and several mint family plants [50]–[52]. Their functionality as a semiochemical varies among different natural systems, including pheromones, kairomones, synomones etc. For example, in the case of decay fungi, C_8_-alcohols may indicate unacceptable hosts or non-hosts as a trophic signal for the conifer feeding bark beetle prey, and as a bypass-trophic signal for the predators.

The checkered beetle *T. formicarius* is known to prey upon 27 bark beetle species in Europe, see [21] and references therein, and can conceivably exploit a broad set of chemical signals from both its prey and prey's host trees [26], [27]. Considering the broad spectrum of chemical signals exploited, *T. formicarius* could be viewed as a generalist predator of bark beetles [53]. However, since this clerid feeds on the patch-scale and habitat-scale within the trees and groups of trees colonized mainly by conifer bark beetles [21], pp 61–64, *T. formicarius* could also be considered to be a “habitat specialist” of coniferous forests [54]. There are observations indicating that this predator could be a habitat specialist of coniferous forest also on the larger of scales of stand or landscape [19]–[21]. Therefore, the recognition of volatiles from trees not exploited by the prey of the clerids (i.e. NHV for the prey) would be adaptive for such “habitat specialist” predators, and would further increase the searching efficacy in the habitat and prey finding process. Interestingly, it has been concluded that both specialist and generalist arthropod carnivores may commonly use attractant semiochemicals in foraging [55] and not only the specialists as previously suggested [2].

The negative effect of odors from unsuitable habitat or plants is better known in another guild of third trophic level insects, the parasitoids. The negative effect of non-host plants of herbivores on the attack rates of two parasitoids, the braconid wasp *Cotesia rubecula* and the tachinid fly *Bessa herveyi*, was observed in two early papers [56], [57]. Powell & Wright [58] indicated that the oviposition rate of a parasitoid, *Aphidius rhopalosiphi* (Hym.: Braconidae) was reduced on a non-preferred host aphid *Acyrthosiphon pisum* in the presence of a non-food plant *Vicia faba*, of its preferred aphid host. Gohole et al. [59] reported a repellent effect of volatiles from the molasses grass (*Melinis minutiflora* [Poaceae]), a non-host plant of the maize stemborer *Chilo partellus* (Lepidoptera: Crambidae) on a pupal parasitoid *Dentichasmias busseolae* (Hym.: Ichneumonidae).

Compared to the parasitoids, less is known about the use of semiochemicals by insect predators in finding the habitat of their prey [2], [3], and very little is known concerning olfactory signals that inhibit attraction. In a field trapping study, Schroeder [6] found that the attraction to ethanol-baited traps of *Rhizophagus depressus* (Col.: Rhizophagidae), a predator species inhabiting the galleries of conifer bark beetles such as *To. piniperda* and *Hylurgops palliatus*, was reduced in the presence of aspen and birch wood. It is still unknown which kind of volatile chemicals from the angiosperm wood was responsible for these inhibitory effects. Recently, two coleopteran predators of conifer bark beetles in North America [*Enoclerus sphegeus*, Cleridae and *Lascontonus tuberculatus*, Colydiidae] were also shown to have repeatable antennal responses to several angiosperm volatiles, NHV for their prey. These included C_8_-alcohols, GLV alcohols, and *trans*-conophthorin [60], which suggest a more widespread perception of specific bypass-trophic signals.

The recognition and orientation of predators to bark beetle aggregation pheromones and to volatiles from the conifer hosts of bark beetles are likely to exert strong selection pressures on the bark beetles. Bark beetles, in turn, have developed strategies to escape from predators without sacrificing the intraspecific functionality of the pheromones [1], such as alternations in pheromone stereochemistry [54], [61], [62], use of additional pheromone components [63], or optimal response to different release rates than the predator [64]. One may speculate that the clear disparity in response to the volatiles from plants not exploited by the prey between prey and predators may provide bark beetles an enemy-free space in some mixed habitats.

Our study demonstrates that the clerid, *T. formicarius*, has evolved the olfactory recognition not only for bark beetle pheromones and host plant volatiles to find their prey [25]–[27], but also for the volatiles from plants not exploited by the prey, probably to avoid searching in the unsuitable patches or habitats. Monoculture stands (habitats) that forest biologists largely study today are mostly a result of one or two centuries of “modern” forest management [19]. The evolution of the sensory apparatus and behavioral responses has taken place in forests of more mixed cover types. Why are both conifer bark beetles and their predators are so sensitive to the volatiles from their non-host and non-prey habitats? Is it a result of ancient host or prey shifts [65], [66] or adaptations to current environments? Phylogenetic analyses of both groups and chemical ecology data on predators of angiosperm bark beetles may shed light on the origin of the high sensitivity to these bi- and bypass-trophic signals (Figure 1).

In a complex environment like a forest, there are probably far more types of chemically detectable volatile molecules present in the air than those involved in the trophic relations (see [5]). Many of them could be expected to act as components of a “background noise” rather than part of a specifically recognized signal [39]. However, our current data suggest that some components of this “background noise” are signals that are specifically detected in the periphery (odorant receptor neurons on the antenna), processed by CNS, and further acted upon by habitat specialist predators and parasitoids as a bypass-trophic signal. Further studies will show if other predators and parasitoids also recognize the volatiles from plants not exploited by the prey (i.e. NHV for the prey or hosts) as specific bypass-trophic semiochemical signals, not as a background noise of many molecules. Based on our current findings and recent reports, we predict that responses to specific bypass-trophic signals will be found in many, if not all, host- or habitat-specific arthropod carnivores.

## Materials and Methods

### Insects

Adult *T. formicarius* were collected in May 1999 from spruce and pine log piles in Asa, Småland, southern Sweden. Adults were maintained separately in Petri dishes with filter paper covering the bottom, and fed with live *I. typographus* adults. Adult *T. formicarius* were kept alive at 4°C until they were used in the electrophysiological and walking bioassay experiments within 1–2 weeks. The collection of predators from mixed conifer log piles may introduce a bias by learning or sub-sampling effects towards a preference of the collected insects for conifer odors in the walking bioassay. Our subsequent field test, however, relied on effects on attraction of a freely dispersing natural population from a forest landscape of both conifer and mixed forests.

### Electrophysiological study

Coupled gas chromatographic-electroantennographic detection (GC-EAD) analyses were carried out on freshly cut antennae by using an HP 6890 gas chromatograph equipped with a fused silica capillary column (HP-Innowax), a 1∶1 effluent splitter that allowed simultaneous flame ionization (FID) and electroantennographic (EAD) detection of the separated volatile compounds [67]. Hydrogen was used as the carrier gas. The column temperature was 40°C for the first 2 min, rising to 200°C via a linear thermal gradient at 10°C min^−1^, and held for 2 min. The outlet for the EAD was inserted into a humidified air-stream (1 L min^−1^) directed over the *T. formicarius* antennal preparation. The freshly cut antenna (basal cut-end) was inserted into a glass capillary indifferent electrode filled with Beadle-Ephrussi Ringer solution, and grounded via a silver wire. A similar recording electrode connected to a high-impedance DC amplifier with automatic baseline drift compensation was placed in contact with the distal end of the antenna (uncut). The antennal signal was stored and analyzed on a PC equipped with an IDAC-card and the program EAD v2.3 (Syntech, Hilversum, The Netherlands). Two similar synthetic kairomone mixtures with 50 ng of each compound passing over the antenna after GC separation were tested against *T. formicarius* antennae (Figure 2): combinations of conifer tree monoterpenes [(±)-α-pinene and Δ^3^-carene)], pheromone components of the *Ips* bark beetle prey [MB, (−)-cV, and (−)-tV), Table 2], non-habitat (non-host) leaf and bark volatiles [(C_6_-alcohols, C_8_-alcohols, and (±)-*trans*-conophthorin)], and (−)-verbenone, which is a well known prey interruptant associated with old, colonized host trees of conifer bark beetles. The doses of chemicals were similar to those used for synthetic blends of NHV tested on scolytid antennae [7], [40], [68]. The synthetic blends were used here, rather than a collection of volatiles from nature, as the full range of inhibitory candidates and attractants of interest for the responses of the predator are not available from any single biological source. Specific data on the commercial sources and chemical and stereochemical purity of each component are provided in Table 2. Each mixture (ca. 1 µl/injection) was tested against five antennae of each sex of *T. formicarius*. A repeatable response was defined as a depolarization of the antennal signal at the same retention time in three of five runs.

**Table 2:** Chemicals, commercial sources, purity, release rates, and dispensers used in laboratory and field studies of the physiology and behavior of the checkered beetle, *Thanasimus formicarius*.

Treatments (signal types)	Chemicals	Source^a^	Purity (%)	Release (mg/day)^b^	Dispensers
***1. Laboratory walking bioassay***					
**Kairomones**					
	2-methyl-3-buten-2-ol (MB)	2	98	2.45	MB and cV at 50∶1 in a 50 µl Microcapsâ^c^
	4S-*cis*-verbenol (cV)	3	97	0.05	
	ipsdienol	2	95	0.04	in 50 µl Microcaps
	(±)-a-pinene	1	98	3	in 50 µl Microcaps
**Prey's nonhost volatiles (NHV)**					
**3GLV:** blend of 3 green leaf alcohols				**0.33**	1∶1∶1 in a 50 µl Microcaps
	1-hexanol	1	98	0.11	
	*Z*-3-hexen-1-ol	1	98	0.11	
	*E*-2-hexen-1-ol	1	97	0.11	
**2C8OH:** blend of 2 bark C8-alcohols				**0.18**	1∶1 in a 50 µl Microcaps
	3-octanol	3	99	0.08	
	1-octen-3-ol	3	98	0.1	
**tC:**	*trans*-conophthorin	4	87	0.12	in 10 µl Microcaps
**Prey's old host signal**					
	(−)-verbenone (**Vn**)	5	99	0.003	added to pheromone neat solution at MB∶cV∶Vn of 50∶1∶0.1 in 50 µl Microcaps
***2. Field flight-trapping assays***					
**Kairomones**					
***Ips typographus*** **pheromone**					
	*cis*-verbenol (cV)	2	97	1	hard PE-vial^d^ with 9-mm-diam. hole in lid
	2-methyl-3-buten-2-ol (MB)	3	98	57	#733 PE-vial^e^ with 2-mm-diam. hole in lid
***Ips duplicatus*** ** pheromone**					
	(±)-ipsdienol	6	95	2.8	SciTech bag dispenser (Praha, Czech Republic)
	*E*-myrcenol	6	95	0.6	
**Monoterpene-mix**				**60**	600 µl of a 2∶1∶1 mix in a closed #733 PE-vial with 6 mm diam. hole in the lid
	(±)-a-pinene	1	98	30	
	3-carene	1	95	15	
	terpinolene	7	85	15	
**Prey's nonhost volatiles (NHV)**					
**3GLV:** blend of 3 green leaf alcohols				**6**	200 µl of a 1∶1∶1 mix in an open #730 PE-vial^f^
	1-hexanol	1	98	2	
	(*Z*)-3-hexen-1-ol	1	98	2	
	(*E*)-2-hexen-1-ol	1	97	2	
**2C8OH:** blend of 2 bark C8-alcohols				**5.6**	2 open #730 PE-vials (200 µl of a 1∶1 mix in each vial)
	(±)-3-octanol	3	99	2.4	
	(±)-1-octen-3-ol	3	98	3.2	
**tC:**					
	*trans*-conophthorin	4	87	5	100 µl in an open # 730 PE-vial
**Prey's old host signal**					
	(−)-verbenone (**Vn**)	5	99	0.5	200 µl in an open #730 PE-vial

a )1: Aldrich, USA; 2: Borregaard, Norway; 3: Acros, USA; 4: Pherotech, CAN; 5: Bedoukian Research INC, USA. 6: SciTech, CZ; 7: C. Roth, Germany.

b )Release rates were estimated by following the retreat of the meniscus over time for capillaries; and measured by weight loss for PE-vials at 20–21°C in lab.

c )Neat compounds evaporating from Microcaps® with one end sealed by dental wax.

d )3 ml-hard polyethylene vial (Kartell, Italy) with 13-mm-diam., 24 mm inner height.

e )Polyethylene vial (Kartell, Italy) with 20-mm-diam., 29 mm inner height.

f )Polyethylene vial (Kartell, Italy) with 6-mm-diam., 29 mm inner height.

### Walking bioassay

Behavioral responses of walking *T. formicarius* were tested in the laboratory by using an open area walking bioassay olfactometer [67], [69]. Bioassays were conducted at 24 to 25°C under 200 lux of white light. The arena (50×50 cm) was swept by laminar airflow at ca. 1 m s^−1^, and an odor plume was generated by placing one or several capillary tubes (50 µl Microcaps® [Drummond Scientific Co., Broomall, PA, USA], inner Ø 0.80 mm) with test materials at the center of the source of the airflow (Figure 3). Walking bioassays were done during daytime; predominantly from 13:00–16:30. Adult *T. formicarius* were randomly grouped (10 beetles/group), and beetles were taken from each group then each released individually downwind in the center of a circle (40 cm ∅) opposite to the odor source. A beetle that walked upwind and reached the source within 1 min were scored as responding. The average response per treatment was calculated on the proportion of the 10 individuals in each group reaching the source, 9 or 14 such groups tested per treatment (total 230 beetles tested). In the positive control, the clerids were tested against a mixture of synthetic kairomone components (Table 2) released from the 50 µL glass capillaries with one end sealed by dental wax. The treatments contained either individual volatiles from plants not exploited by the prey or blends (see Table 2 for details) dispensed in separate capillary tubes, placed at the odor source in contact with the kairomone dispensers. The dose-levels used were similar to those in earlier studies on scolytid NHV [67], [69]. Verbenone (Vn) was tested at a low dose by adding it to the neat kairomone solution at MB∶cV∶Vn of 50∶1∶0.1 (Table 2).

### Field trapping

Two field-trapping experiments were carried out in May–June 2000 in Asa, Småland, Sweden, in the same area where *T. formicarius* adults were collected in 1999 for the electrophysiological studies and walking bioassay experiments. A pair of funnel trap groups, each consisting of a combination of a 12-unit multiple funnel trap (Pherotech (now Contech) Inc., Delta, British Columbia, Canada) and an adjacent a cross barrier trap (Fytofarm Ltd., Bratislava, Slovakia), was set up 10 m apart in a new clear-cut area of mixed spruce and pine forest. To prevent the escape of captured clerids, a dental cotton roll loaded with insecticide (Permethrin) was placed in each trap collector. To minimize positional effects, dispenser positions were switched after each replicate when >10 clerids were caught in either trap group.

Experiment 1 tested the potential inhibitory effect of a blend of the two highly EAD-active C_8_-alcohols [(±)-3-octanol+(±)-1-octen-3-ol]. Traps were baited with kairomone alone (as positive control) or kairomone plus a blend of the two laboratory active C_8_-alcohols (Table 2). The kairomone consisted of the aggregation pheromone components (MB and cV, from *I. typographus* [24], and (±)-ipsdienol and *E*-myrcenol, from *I. duplicatus* [70]) combined with conifer monoterpenes (α-pinene, terpinolene, Δ^3^-carene, an attractant blend for *Tomicus* spp. bark beetles [30], [71]). After six replicates, the same set of traps was used for Experiment 2 with a similar protocol, to test a blend of the weakly EAD-active compounds: three green leaf volatile C_6_-alcohols [GLV: 1-hexanol, (*Z*)-3-hexen-1-ol, and (*E*)-2-hexen-1-ol], (±)-*trans*-conophthorin, and (−)-verbenone (Table 2). Doses of synthetics used were the same as, or similar to, those used in earlier tests for clerid kairomone [24], [53], scolytid host [71] or non-host [7], [9], [10], [67], [72] volatiles.

### Statistical analyses

Data from the laboratory walking bioassay experiments, i.e., proportion (*p*) of clerid beetles responding to the test mixtures, were analyzed by ANOVA of arcsin √*p* followed by comparison of means at α = 0.05 with the REGW-Q post-hoc multiple range test [73]. Due to the zero responses to the blank control and the C_8_-alcohols when tested alone, these two treatments were not included in the ANOVA or in the range tests to achieve homogeneity of variances [74]. The trap catches from field tests were compared by using a paired *t*-test with an experiment-wise α = 0.05. The standardized effect sizes [29] were calculated for the behavioral responses to allow us to compare the effects of the NHV components (for the prey) on laboratory and field responses in the clerid as well as to compare the response in the two field tests that were run at different population levels. The effect size measure scales the difference of means (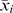) by division of their pooled standard deviations (*SD_i_*) [29]. We used the conservative Hedges *d* measure of effect size [75], [76], which adjusts for sample sizes (*n_i_*) that are low and/or unequal.
